# Self-Rated Health and Sick Leave among Nurses and Physicians: The Role of Regret and Coping Strategies in Difficult Care-Related Situations

**DOI:** 10.3389/fpsyg.2017.00623

**Published:** 2017-04-20

**Authors:** Stéphane Cullati, Boris Cheval, Ralph E. Schmidt, Thomas Agoritsas, Pierre Chopard, Delphine S. Courvoisier

**Affiliations:** ^1^Quality of Care Service, University Hospitals of GenevaGeneva, Switzerland; ^2^Department of General Internal Medicine, Rehabilitation and Geriatrics, University of GenevaGeneva, Switzerland; ^3^Swiss NCCR “LIVES: Overcoming Vulnerability: Life Course Perspectives”, University of GenevaGeneva, Switzerland; ^4^Institute of Sociological Research, University of GenevaGeneva, Switzerland; ^5^Department of Psychology, University of GenevaGeneva, Switzerland; ^6^Methodology and Data Analysis Laboratory, University of GenevaGeneva, Switzerland; ^7^Division of General Internal Medicine, University Hospitals of GenevaGeneva, Switzerland; ^8^Division of Clinical Epidemiology, University Hospitals of GenevaGeneva, Switzerland; ^9^Department of Clinical Epidemiology and Biostatistics, McMaster University, Faculty of Health Sciences, HamiltonON, Canada

**Keywords:** self-rated health, sick leave, moral distress, healthcare-related regrets, nurses, physicians

## Abstract

Moral distress – such as feeling strong regret over difficult patient situations – is common among nurses and physicians. Regret intensity, as well as the coping strategies used to manage regrets, may also influence the health and sickness absence of healthcare professionals. The objective of this study was to determine if the experience of regret related to difficult care-related situations is associated with poor health and sick leave and if coping strategies mediate these associations. Two cross-sectional surveys were conducted in Switzerland (Geneva, 2011 and Zurich, 2014). Outcomes were self-rated health (SRH) and sick leave in the last 6 months. We examined the associations of regret intensity with the most important care-related regret, number of recent care-related regrets, and coping strategies, using regressions models. Among 775 respondents, most reported very good SRH and 9.7% indicated absence from work during four working days or more. Intensity of the most important regret was associated with poor SRH among nurses and physicians, and with higher sick leave among nurses. Maladaptive emotion-focused strategies were associated with poor SRH among nurses, whereas adaptive emotion-focused strategies were positively associated with higher SRH and lower sick leave among physicians. Because care-related regret is an integral part of clinical practice in acute care hospitals, helping physicians and, especially, nurses to learn how to deal with negative events may yield beneficial consequences at the individual, patient care, and institutional level.

## Introduction

Providing safe and good quality healthcare to patients in acute care settings requires healthy healthcare professionals (such as physicians and nurses) (Rowe and Kidd, 2009; West et al., 2009). Yet, many studies have documented that healthcare professionals’ own health is often far from optimal. Compared to the general population, healthcare professionals more frequently report poor self-rated health (SRH) (Knecht et al., 2010; Malinauskiene et al., 2011), sleeping problems or poor sleep quality (Aasa et al., 2005; Hsieh et al., 2011; Bjorvatn et al., 2012; Ghalichi et al., 2013), and suffer more often from depression (Embriaco et al., 2012) or burn out (Embriaco et al., 2007; Arigoni et al., 2010; Merlani et al., 2011). The economic burden of these health-related problems is high for institutions (Schultz et al., 2009; Letvak et al., 2012).

Similarly, moral distress is also common among healthcare professionals working in acute care settings (Pauly et al., 2009; Sirriyeh et al., 2010; Huffman and Rittenmeyer, 2012; Burston and Tuckett, 2013; Oh and Gastmans, 2015). Moral distress can be related to the healthcare professionals’ negative judgment about the quality of care he/she provided to the patients, such as inappropriate care (Piers et al., 2011), loss of control (Shapiro et al., 2011) or stress of conscience (Glasberg et al., 2008), or to healthcare professionals’ involvement in medical errors (Sirriyeh et al., 2010). One emotional facet of moral distress is the experience of regret (Zeelenberg and Pieters, 2007). Healthcare professionals may regret the care that they provided when they feel that it was inconsistent with their personal beliefs or clinical knowledge (Courvoisier et al., 2011, 2013b). The experience of care-related regret is frequent (Courvoisier et al., 2013a), in part because working in acute care hospitals constrains the way healthcare professionals make clinical decisions and attend to patients’ care. The hospital setting entails high workload (Hugonnet et al., 2007; Weissman et al., 2007), shift work, information overload (Rössler, 2012), and the daily confrontation with numerous complex and uncertain clinical situations (Thompson and Dowding, 2001; Nevalainen et al., 2013). Experiencing regrets, may be associated with poor health conditions among healthcare professionals (Schmidt et al., 2015).

**FIGURE 1:**
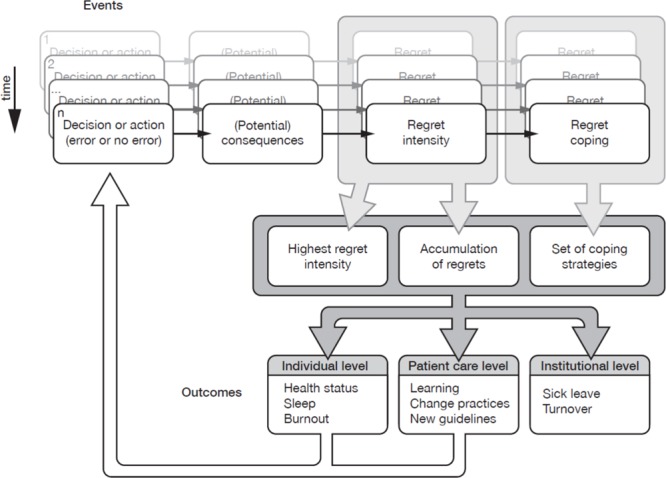
**Theoretical framework of experiencing regret in clinical practice and its implications.** Lancet (Courvoisier et al., 2013b).

Conceptually, three aspects of care-related regret may influence health: the intensity of the most important regret, the accumulation of small regrets experienced throughout the daily working time, and the strategies used to cope with daily regrets (**Figure 1**) (Courvoisier et al., 2013b). The strategies can be further categorized into three main types: problem-focused strategies (e.g., talking to the patient), emotion-focused adaptive strategies (e.g., accepting one’s own limitations or the inherent limitations of medicine), and emotion-focused maladaptive strategies (e.g., ruminating the events and possible implications). A systematic review showed that adaptive strategies are frequent when healthcare professionals are facing patient adverse events (Seys et al., 2013). Although adaptive strategies may reduce the burden of regret intensity and regret accumulation on health (DeSteno et al., 2013), no study has yet examined specific mechanisms underlying the associations.

The objectives of the present study conducted among hospital-based nurses and physicians were therefore to assess whether their health conditions (SRH and self-reported absenteeism) are associated with (1) care-related regrets (intensity of most important regret, accumulation of regrets) and regret coping strategies; and (2) to test whether coping strategies mediated the associations between care-related regrets and health conditions.

## Materials and Methods

### Study Design

A first survey was conducted in 2011 at the University Hospitals of Geneva, a 1800-beds Swiss public hospital network. Mail questionnaires were sent to 825 nurses and 825 physicians with up to three reminders. A second survey was conducted in 2014 at the Stadtspital Triemli, Zurich (500-beds hospital) and at the Bezirksspital Affoltern am Albis (100-beds hospital). In this second survey, participants were informed that a small incentive for each completed questionnaire was donated to the Children’s Charity “Theodora” (“Giggle doctors,” Aargau, Switzerland). Physicians or nurses were excluded from these studies if they did not practice in the past 5 years, were retired, or reported the absence of any regrets. The World Medical Association’s Declaration of Helsinki was followed in the conduct of the study. The Research Ethics Committee of the University Hospitals of Geneva and the Ethics Committee Zurich indicated that both surveys were exempted from formal research ethics approval.

### Outcomes

SRH was measured by the first question of the Short Form (36) Health Survey. Following the stem “In general, do you think your health is…,” participants were asked to rate their health as bad (1), fair (2), good (3.7), very good (4.5), or excellent (5). This coding scheme (1, 2, 3.7, 4.5, and 5) was used to better capture the underlying health construct, since the magnitudes between the response options are not evenly distributed as an interval variable (Perneger et al., 2013). SRH is regularly used in health surveys because it measures physical, mental, and social dimensions of health (Singh-Manoux et al., 2006; Perruccio et al., 2012), and predicts several health outcomes, such as sick leave (Halford et al., 2012) and mortality (Aichele et al., 2016).

Sick leave was measured with a question asking respondents the number of workdays they missed in the last 6 months, excluding leaves related to pregnancy and maternity. This variable was dichotomized at the 75th percentile for each profession, that is 0 day versus 1 day or more for physicians, and 0–3 days versus 4 or more days for nurses.

### Independent Variables

*The intensity of the most important care-related regret* was assessed with the 10-item regret intensity scale (RIS-10) (Courvoisier et al., 2013a). The regret must be related to providing healthcare to a patient, and experienced less than 5 years ago. Items are assessed with a five-point Likert scale ranging from “not at all” to “absolutely.” The RIS-10 possesses good psychometric properties, with an internal consistency of 0.87 (Cronbach’s alpha) and a test–retest reliability of 0.70. In addition to the RIS-10, one question probed whether the respondent felt that the event eliciting their most important regret was related to an error (“According to you, did this event imply an error on your part” answered yes or no).

*Care-related regret coping* was assessed with the 15-item regret coping scale (RCS-HCP) (Courvoisier et al., 2014), developed to assess daily coping strategies used by healthcare professionals to deal with regrets they feel when providing care to patient. Using a four-point Likert scale ranging from 1 (“never or almost never”) to 4 (“always or almost always”), RCS-HCP measures how frequently the respondent uses the three types of regret regulation strategies: problem-focused strategies, emotion-focused adaptive strategies, and emotion-focused maladaptive strategies. The three subscales of RCS-HCP have good psychometric properties, with internal consistencies close to 0.90 (Cronbach’s alpha) and test–retest agreement ranging from 0.78 to 0.82 (Courvoisier et al., 2014).

*Recent care-related regrets* were assessed using two questions: “Within these last 30 working days, how many situations with patients have you regretted?” and “What is the mean intensity you would give to these situations within the last 30 days?,” on a visual analog scale from 0 to 10. If the respondent reported no regret in the last 30 days, the mean intensity was imputed as 0.

*Professional and socio-demographic items* were assessed with questions including gender, age, profession (physician or nurse), supervising role (yes, no), years of clinical experience, and percentage of clinical activity (0–50, 51–80, >80%).

### Analysis

The associations of the three care-related variables (i.e., current intensity of the most important regret in the last 5 years, whether the most important regret was an error, number of recent regrets and regret coping) with SRH were examined using linear regression for the SRH score and logistic regression for sick leave. For each outcome, we ran two types of multivariable models. In the first type, we adjusted the analysis of each variable related to regret with the socio-professional variables: gender, years of experience, professional status, percentage of clinical activity, and linguistic region of the hospital. Age was not included because it was too strongly related with years of experience. In the second type of multivariable models, all variables pertaining to care-related regrets and all socio-professional variables were included. All analyses were conducted separately for nurses and physicians. Mediation analyses were performed using Sobel tests with bootstrap confidence intervals. Specifically, the Sobel test compares the coefficient of each regret variable in a model adjusted for socio-professional variables (the corresponding coefficient is called c) to the coefficient of the same regret variable in the model adjusted for socio-professional variables and a regret coping strategy (the corresponding coefficient is called c′). Mediation analyses tested each dimension of coping separately and the three dimensions together. All analyses were conducted with R (version 3.2.4, Vienna, Austria).

We further conducted two sensitivity analyses. First, we ran the same analyses on all healthcare professionals, without stratifying on profession (nurses and physicians together). Second, we examined others ways to model SRH than as a continuous variable with evenly spaced ratings values (1, 2, 3.7, 4.5, and 5) (Perneger et al., 2013), such as an interval scale or a dichotomized scale between very good or excellent versus less than very good.

## Results

Overall, 775 participants returned the survey (22.5% of the 3,452 eligible participants). Healthcare professionals had a mean age of 39.5 (*SD*: 9.1) years. The majority were women, nurses, and had a percentage of clinical activity between 80 and 100% (**Table 1**). Mean number of years in clinical practice was 15 (median: 12, *SD*: 10). Most healthcare professionals reported very good SRH status, physicians more than nurses (*p* < 0.001). Prolonged sick leaves (≥10 days) were reported by 1.7% of physicians compared to 6.3% of nurses (*p* < 0.001).

**Table 1:** Characteristics of healthcare professionals.

		Nurses		Physicians	
Characteristics	Categories	*N* = 466		*N* = 309	
*Socio-professional:*		*N*	%	*N*	%
Gender	Male	64	13.9	138	44.7
	Female	398	86.1	171	55.3
Age	<30	77	16.7	65	21.2
	30–39	157	34.0	144	47.1
	40–49	133	28.8	69	22.6
	>50	95	20.6	28	9.2
Professional status	Nurse/resident	395	86.2	141	45.8
	Head nurse/board certified	63	13.8	167	54.2
Percentage of clinical activity	0–50%	35	7.5	10	3.3
	51–80%	174	37.5	40	13.1
	81–100%	255	55.0	256	83.7
Years of experience	<3	30	6.5	30	9.9
	3–5	26	5.6	77	25.3
	6–10	73	15.8	81	26.6
	11–20	148	32.0	74	24.3
	>20	185	40.0	42	13.8
Hospital’s linguistic region	French	249	53.4	220	71.2
	German	217	46.6	89	28.8

*Care-related regrets:*					
	Mean	SD	Mean	SD
Current intensity of the most important regret experienced in the last 5 years (RIS-10)	1.86	0.72	1.81	0.76
Number of regrets (last month)	2.36	15.4	1.00	1.5
Mean intensity of regrets (last month)	2.35	2.57	2.06	2.36
Daily regret coping strategies:	Problem-focused	2.86	0.61	2.95	0.58
(RCS-HCP)	Maladaptive	1.78	0.57	1.87	0.71
	Adaptive	2.60	0.54	2.73	0.58
	*N*	%	*N*	%
The most important regret in the last 5 years perceived as an error	119	26.0	140	45.9

*Health outcomes:*					
Self-rated health	Bad	1	0.2	0	0.0
	Fair	29	6.3	14	4.5
	Good	180	38.9	60	19.4
	Very good	173	37.4	124	40.1
	Excellent	80	17.3	111	35.9
Sick leave	0 days	301	65.3	239	77.4
	1–3 days	101	21.9	56	18.1
	4–5 days	14	3.0	4	1.3
	6–10 days	16	3.5	5	1.6
	≥10 days	29	6.3	5	1.6

### Care-Related Regret and Health Conditions among Nurses (**Table 2**)

**Table 2:** Association of care-related regret with health conditions among nurses.

	Adjusted analyses, regret variables not included^∗^			Adjusted analyses, all regret variables included^∗^		
Self-rated health¶	Slope	*p*	95% CI	Slope	*p*	95% CI
Most important care-related regret in the last 5 years						
Current intensity (RIS-10)	–0.19	<0.001	–0.29; -0.09	–0.15	0.04	–0.25; -0.02
Self-perceived error	–0.12	0.26	–0.34; 0.09	0.03	0.84	–0.23; 0.33
Recent care-related regrets (last 30 working days)						
Number of regrets	–0.01	0.74	–0.08; 0.06	–0.03	0.48	–0.09; 0.05
Mean intensity	–0.04	0.44	–0.15; 0.06	–0.00	0.99	–0.12; 0.10
Care-related regret coping (RCS-HCP)						
Problem-focused	–0.03	0.55	–0.12; 0.06	–0.01	0.88	–0.14; 0.08
Adaptive emotion-focused	0.04	0.39	–0.05; 0.14	0.03	0.61	–0.09; 0.15
Maladaptive emotion-focused	–0.24	<0.001	–0.34; -0.14	–0.15	0.04	–0.31; -0.03

**Sick leave^x^**	OR	*p*	95% CI	OR	*p*	95% CI
Most important care-related regret in the last 5 years						
Current intensity (RIS-10)	1.34	0.046	1.00; 1.78	1.84	0.005	1.21; 2.86
Self-perceived error	1.14	0.71	0.55; 2.25	0.97	0.94	0.39; 2.24
Recent care-related regrets (last 30 working days)						
Number of regrets	1.03	0.82	0.65; 1.22	1.04	0.76	0.57; 1.27
Mean intensity	1.11	0.46	0.84; 1.47	1.05	0.73	0.77; 1.43
Care-related regret coping (RCS-HCP)						
Problem-focused	0.96	0.77	0.71; 1.29	1.01	0.96	0.71; 1.44
Adaptive emotion-focused	1.12	0.48	0.82; 1.51	1.37	0.10	0.95; 2.00
Maladaptive emotion-focused	1.13	0.46	0.81; 1.54	0.93	0.75	0.60; 1.41

*^∗^For gender, years of experience, professional status, percentage of clinical activity, and linguistic region of the hospital.*

*¶Considered as continuous (Perneger et al., 2013): 1 = bad, 2 = fair, 3.7 = good, 4.5 = very good, 5 = excellent.*

*^x^In the last 6 months. This variable was dichotomized at the 75th percentile for each profession (4 days or more for nurses and 1 day or more for physicians).*

Adjusted analyses examining each regret variables separately (i.e., intensity of the most important regret, self-perceived error of the most important regret, number and intensity of recent regrets, regret coping) showed that higher intensity of the most important regret was associated with poor SRH and sick leave, and that more frequent use of maladaptive coping strategies was associated with poor SRH. Perceiving the most important regret as an error and recent regrets experienced in the previous 30 working days (number of regrets and mean intensity) were not associated with health conditions.

In adjusted models including all regret variables, intensity of the most important regret and more frequent use of maladaptive coping strategies remained associated with poor health conditions. Analysis of the mediation effects of regret coping strategies revealed that maladaptive emotion-focused coping partly mediated the effect of intensity of the most important regret (c–c′ estimate: -0.05, bootstrap 95%CI: [-0.10; -0.01]) on SRH only, while problem-focused and adaptive strategies had no mediation effect (Supplementary Material).

### Care-Related Regret and Health Conditions among Physicians (**Table 3**)

**Table 3:** Association of care-related regret with health conditions among physicians.

	Adjusted analyses, regret variables not included^∗^			Adjusted analyses, all regret variables included^∗^		
Self-rated health¶	Slope	*p*	95% CI	Slope	*p*	95% CI
Most important care-related regret in the last 5 years						
Current intensity (RIS-10)	–0.19	<0.001	–0.30; -0.08	–0.15	0.07	–0.32; 0.01
Self-perceived error	0.08	0.50	–0.14; 0.29	0.12	0.34	–0.13; 0.36
Recent care-related regrets (last 30 working days)						
Number of regrets	–0.81	0.06	–1.66; 0.04	–0.11	0.83	–1.15; 0.93
Mean intensity	–0.20	0.002	–0.32; -0.07	–0.10	0.18	–0.26; 0.05
Care-related regret coping (RCS-HCP)						
Problem-focused	0.02	0.74	–0.10; 0.13	–0.07	0.27	–0.21; 0.06
Adaptive emotion-focused	0.17	0.002	0.06; 0.27	0.15	0.02	0.02; 0.27
Maladaptive emotion-focused	–0.12	0.01	–0.22; -0.03	–0.01	0.83	–0.15; 0.12
**Sick leave^x^**	OR	*p*	95% CI	OR	*p*	95% CI
Most important care-related regret in the last 5 years						
Current intensity(RIS-10)	0.97	0.83	0.72; 1.27	0.75	0.21	0.48; 1.16
Self-perceived error	1.21	0.51	0.69; 2.11	1.21	0.54	0.65; 2.25
Recent care-related regrets (last 30 working days)						
Number of regrets	3.31	0.24	0.41; 24.51	5.61	0.17	0.45; 64.7
Mean intensity	1.04	0.80	0.76; 1.41	1.05	0.82	0.69; 1.55
Care-related regret coping (RCS-HCP)						
Problem-focused	1.06	0.70	0.79; 1.43	1.19	0.30	0.85; 1.68
Adaptive emotion-focused	0.76	0.046	0.57; 0.99	0.67	0.01	0.48; 0.92
Maladaptive emotion-focused	1.03	0.81	0.80; 1.31	0.97	0.88	0.67; 0.39

*^∗^For gender, years of experience, professional status, percentage of clinical activity, and linguistic region of the hospital.*

*¶Considered as continuous (Perneger et al., 2013): 1 = bad, 2 = fair, 3.7 = good, 4.5 = very good, 5 = excellent.*

*^x^In the last 6 months. This variable was dichotomized at the 75th percentile for each profession (4 days or more for nurses and 1 day or more for physicians).*

Adjusted analyses examining regret variables separately showed that higher level in intensity of the most important regret and of recent regrets were associated with poor SRH. More frequent use of adaptive coping strategies was associated with good SRH and with lower probability of sick leave, while more frequent use of maladaptive strategies was associated with poor SHR. As in the sample of nurses, perceiving the most important regret as an error was not associated with health conditions.

In adjusted models including all regret variables, only more frequent use of adaptive coping strategies remained associated with health conditions, while factors like intensity of the most important regret and of recent regrets were no longer associated with SRH. Analysis of the mediation effects of regret coping strategies revealed that more frequent use of adaptive emotion-focused strategies had an indirect, protective, effect in three associations: between recent regrets and sick leave (c–c′ estimate: -0.58, bootstrap 95%CI: [-2.82; -0.08]) and between the most important regret and SRH (0.08, [0.02; 0.18]) and sick leave (0.08, [0.01; 0.17]) (Supplementary Material).

### Sensitivity Analysis

Analyses without stratifying on profession (nurses and physicians together) yielded similar results. When SRH was dichotomized, the only significant predictors of SRH were adaptive strategies for physicians (OR = 0.64, 95%CI: [0.46; 0.86]) and maladaptive strategies for nurses (OR = 1.42, 95%CI: [1.10; 1.85]). By contrast, when SRH was treated as an interval scale results were similar to the previous ones.

## Discussion

The experience of care-related regrets — an emotional facet of moral distress — seems to be an unavoidable corollary of acute care practice, which is characterized by time pressure (Weissman et al., 2007), information overload and the increasing complexity and uncertainty related to patient care (Nevalainen et al., 2013). Moreover, suboptimal regret coping strategies may have a negative impact on clinicians’ health and, eventually, on the quality of care they provide to patients (Rowe and Kidd, 2009; West et al., 2009). The aim of this study was twofold: first, to assess the cross-sectional associations between care-related regret experiences and health conditions; second, to test whether coping strategies mediated the relationships between care-related regrets and health conditions.

### Care-Related Regrets

Results revealed that higher level of intensity of the most important regret in the previous 5-years was associated with poor SRH among both nurses and physicians, and with higher sick leave among nurses. In accordance with our proposed theoretical model (Courvoisier et al., 2013b), these findings suggest that lingering feelings of moral distress regarding perceived shortcomings or failures in patient care may impact healthcare professionals’ own health in important ways. These findings are also in line with a recent systematic review pointing out that even if distressing experiences are not very frequent among nurses, their impact can be significant a long time after the event (Oh and Gastmans, 2015), a phenomenon which has previously been labeled “moral residue” (Webster and Baylis, 2000). In contrast, the mean intensity of recent regrets (i.e., within the previous 30 working days) was only associated with poor health among physicians. This result is in line with a so-called “crescendo effect” resulting from repetitive exposure to morally distressing situations with patients (Epstein and Hamric, 2009).

### Care-Related Regret Coping Strategies

Our findings showed that maladaptive emotion-focused strategies were associated with poor SRH among nurses. This effect remained significant in the model that included all regret and socio-professional variables, in contrast to the corresponding analyses for physicians. Another difference between nurses and physicians emerged regarding the use of adaptive emotion-focused strategies: while it was positively associated both with higher SRH and lower sick leave among physicians, it was not significantly related to these variables among nurses. The positive association between adaptive regret coping strategy and physicians’ health is in line with a systematic review indicating that medical errors do not only entail negative consequences, but may also result in positive outcomes related to psychological well-being and improved teamwork collaboration (Sirriyeh et al., 2010). Interestingly, physicians seem better positioned than nurses to cope with negative events or unsatisfactory patient care quality, perhaps because they feel more in charge of key elements of clinical decision making. Having such efficient coping skills is susceptible to explain, at least in part, why they reported better SRH status and fewer sick leave days, when compared with nurses. An alternative explanation is that it may be more difficult for nurses to take emotional distance because they tend to have more frequent and close interactions with patients at their bedside.

Moreover, recent research suggests that coping strategies are not adaptive or maladaptive *per se*; rather the adaptiveness depends on the context. For instance, reappraisal may be adaptive when stressors are uncontrollable (when the person can regulate only the self) but maladaptive when stressors can be controlled (when the person can change the situation) (Troy et al., 2013). In situations when nurses have an inferior status in the hospital hierarchy, they may often renounce external problem-focused coping and instead turn to internal emotion-focused strategies, which may prove maladaptive in that they do not improve working conditions.

### The Meditational Role of Coping Strategies

Irrespective of the objective incidence of difficult situations, learning how to effectively manage such situations may modulate their impact on one’s health. In the clinical context, the use of adaptive and maladaptive coping strategies may help explain why similar distressing experiences occurring in patient care can entail either positive or negative consequences for healthcare professionals’ own health (Courvoisier et al., 2011). Among nurses, however, there was no evidence suggesting that problem-focused and adaptive coping strategies mediated the relationship between care-related regrets and SRH.

In contrast, maladaptive coping strategies seemed to mediate the relationship between care-related regrets and SRH, suggesting that nurses’ use of maladaptive strategies increases the burden associated with their most important care-related regret. This finding concurs with empirical observations that intense and frequent regrets tend to elicit the use of maladaptive strategies (Sheppes et al., 2011; Courvoisier et al., 2014).

A plausible hypothesis may well be that problem-focused and adaptive strategies are only used up to a certain level of regret; once that level is reached, people typically turn to maladaptive strategies. Nurses’ perception that they lack autonomy at work may also be an additional explanatory factor (Papathanassoglou et al., 2012). In contrast, physicians’ use of coping strategies mediated the effect of the number of recent healthcare-related regrets on sick leave. The use of adaptive emotion-focused coping strategies mediated the effect of the most important regret on both SRH and sick leave. To sum up, the present study provided only partial evidence for a mediating role of coping strategies, especially among nurses for whom it appears that the coping strategies exert a direct influence on their health. Programs that help healthcare professionals cope with care-related situations, like clinical supervision, debriefings and Balint groups, should be expanded for the benefit of clinicians’ own well-being.

### Study Limitations

The strengths of the current study include: (a) a relatively large sample size and recruitment in three hospitals in two different linguistic regions; (b) the use of validated scales; and (c) the inclusion of both nurses and physicians, which increases the generalizability of our results to healthcare professionals working in acute care hospitals. Nevertheless, at least three limitations have to be noted. First, our survey obtained a low response rate. Such response rates are not rare in health services research (McLeod et al., 2013), particularly on sensitive topics, such as distress, but it raises concerns about selection bias when estimating prevalence of health conditions. Despite this limitation on absolute estimates, the associations found in this study are quite congruent with the expected results (e.g., negative association of maladaptive regret coping with health). As a result, it seems rather unlikely that the associations of regret with health that we observed should differ between non-respondents and respondents. Second, because SRH has been shown to vary by race (Landrine et al., 2016), the current results may not generalize to all races. Third, we did not adjust for some potential confounders, including night shifts, a factor that could moderate the association between regret and health. Third, due to the cross-sectional design of this study, we cannot determine the causal nature of the associations between regret and health, although the use of adjustment for socio-professional variables should limit the impact of potential confounds.

## Conclusion

Because care-related regret is an inevitable part of clinical practice, healthcare professionals need to cope with such negative feelings. When effective, these coping skills may have the potential not only to protect healthcare professionals’ own health, but also to promote better quality of care (Burston and Tuckett, 2013). Our findings suggest that physicians may be better positioned than nurses to effectively cope with negative events, notably those that were causing stronger distress. Helping healthcare professionals, especially nurses, to adopt and implement effective coping strategies in their daily practice may produce beneficial consequences at the individual, patient care, and institutional level.

## Author Contributions

DC and SC conceived the research idea and design. SC, BC, RS, TA, PC, and DC contributed for preparing and revising the draft. SC, BC, and DC participated in the analysis of the data and were responsible for the preparation of the article.

## Conflict of Interest Statement

The authors declare that the research was conducted in the absence of any commercial or financial relationships that could be construed as a potential conflict of interest.
