# Supplementary material for: Self-Rated Health and Sick Leave among Nurses and Physicians: The Role of Regret and Coping Strategies in Difficult Care-Related Situations
Source: Front Psychol. 2017 Apr 20;8:623. doi: 10.3389/fpsyg.2017.00623 (PMC5397490; doi:10.3389/fpsyg.2017.00623)
Supplement: Supplementary file 1 [file Table_S1.DOCX]

Supplementary Material

**Self-rated health and sick leave among nurses and physicians: the role of regret in difficult care-related situations**

Stéphane Cullati, PhD*^1-4^, Boris Cheval, PhD*^1, 2, 5^, Ralph E. Schmidt, PhD^5^, Thomas Agoritsas, MD^6-8^, Pierre Chopard, MD^1, 2^, Delphine S. Courvoisier, PhD^1, 2^

* Both authors contributed equally

**Supplementary material**

Mediation analyses: the Sobel test compares the coefficient of each regret variable in a model adjusted for socio-professional variables (the corresponding coefficient is called c) to the coefficient of the same regret variable in the model adjusted for socio-professional variables and a regret coping strategy (the corresponding coefficient is called c’).

Table S1. Mediation between care-related regrets and self-rated health^¶^ and sick leave^§^ in the last 6 months.

|  |  | Self-rated health | | Sick leave | |
| --- | --- | --- | --- | --- | --- |
|  | Mediator | c-c’ | Bca 95%CI | c-c’ | Bca 95%CI |
| **Nurses** |  |  |  |  |  |
| RIS | PF | 0.01 | -0.00; 0.03 | -0.03 | -0.12; 0.01 |
| RIS | A | 0.01 | -0.01; 0.03 | -0.08 | -0.19; 0.02 |
| RIS | MA | -0.05* | -0.10; -0.01 | -0.08 | -0.29; 0.12 |
| RIS | All strategies | -0.05* | -0.10; -0.01 | -0.12 | -0.35; 0.10 |
| Recent regrets | PF | 0.00 | -0.00; 0.01 | -0.001 | -0.02; 0.02 |
| Recent regrets | A | -0.00 | -0.01; 0.00 | -0.01 | -0.06; 0.01 |
| Recent regrets | MA | 0.00 | -0.01; 0.02 | -0.01 | -0.06; 0.02 |
| Recent regrets | All strategies | 0.00 | -0.01; 0.02 | -0.02 | -0.08; 0.04 |
| **Physicians** |  |  |  |  |  |
| RIS | PF | 0.02 | -0.06; 0.05 | 0.002 | -0.06; 0.04 |
| RIS | A | 0.08* | 0.02; 0.18 | 0.08* | 0.01; 0.17 |
| RIS | MA | -0.01 | -0.09; 0.06 | 0.08 | -0.14; 0.33 |
| RIS | All strategies | -0.03 | -0.11; 0.04 | 0.13 | -0.12; 0.39 |
| Recent regrets | PF | -0.05 | -0.62; 0.05 | -0.29 | -2.11; 0.02 |
| Recent regrets | A | 0.04 | -0.19; 0.36 | -0.58* | -2.82; -0.08 |
| Recent regrets | MA | -0.04 | -0.54; 0.08 | -0.24 | -1.90; 0.07 |
| Recent regrets | All strategies | 0.07 | -0.17; 0.48 | - -0.63* | -2.90; -0.05 |

* means p<0.05.

^¶^ considered as continuous([Perneger et al. 2013](#_ENREF_29)): 1=bad, 2=fair, 3.7=good, 4.5=very good, 5= excellent

^§^ this variable was dichotomized at the 75^th^ percentile for each profession

Abbreviations: RIS= Most important care-related regret in the last 5 years (RIS-10); Recent regrets = number of care-related regrets in the last 30 working days; PF= problem-focused strategies, A= adaptive emotion-focused strategies, MA= maladaptive emotion-focused strategies. Bca = bias-corrected acceleration 95% confidence intervals
